# Longitudinal Analysis of Real-World Performance of an Implantable Continuous Glucose Sensor over Multiple Sensor Insertion and Removal Cycles

**DOI:** 10.1089/dia.2019.0342

**Published:** 2020-04-27

**Authors:** Katherine S. Tweden, Dorothee Deiss, Ravi Rastogi, Suresh Addaguduru, Francine R. Kaufman

**Affiliations:** ^1^ Senseonics, Incorporated, Germantown, Maryland.; ^2^ Center for Endocrinology and Diabetology, Medicover Berlin-Mitte, Berlin, Germany.

**Keywords:** Continuous glucose monitoring, Sensor, Implantable, Type 1 diabetes, Type 2 diabetes, CGM metrics, Glucometrics, Accuracy

## Abstract

The Eversense^®^ Continuous Glucose Monitoring (CGM) System, with the first long-term, implantable glucose sensor, has been commercially available in Europe and South Africa since 2016 for adults with diabetes. The performance of the sensor over multiple, sequential 90- or 180-day cycles from either real-world experience or clinical studies has not been previously published. The Eversense Data Management System (DMS) was used to evaluate the accuracy of General Data Protection Regulation (GDPR)-compliant sensor glucose (SG) values against self-monitoring of blood glucose (SMBG) from June 2016 through August 2019 among patients with at least four sensor cycles from European and South African health care practices. Mean SG and associated measures of variability, glucose management indicator (GMI), and percent and time in various hypoglycemic, euglycemic, and hyperglycemic ranges were calculated for the 24-h time period over each cycle. In addition, transmitter wear time was evaluated across each sensor wear cycle. Among the 945 users included in the analysis, the mean absolute relative difference (standard deviation [SD]) using 152,206, 174,645, 206,024, and 172,587 calibration matched pairs against SMBG was 11.9% (3.6%), 11.5% (4.0%), 11.8% (4.7%), and 11.5% (4.1%) during the first four sensor cycles, respectively. Mean values of the CGM metrics over the first sensor cycle were 156.5 mg/dL for SG, 54.7 mg/dL for SD, 0.35 for coefficient of variation, and 7.04% for GMI. Percent SG at different glycemic ranges was as follows: <54 mg/dL was 1.1% (16 min), <70 mg/dL was 4.6% (66 min), ≥70–180 mg/dL (time in range) was 64.5% (929 min), >180–250 mg/dL was 22.8% (328 min), and >250 mg/dL was 8.1% (117 min). The median transmitter wear time over the first cycle was 83.2%. CGM metrics and wear time were similar over the subsequent three cycles. This real-world evaluation of adult patients with diabetes using the Eversense CGM System in the home setting demonstrated that the implantable sensor provides consistent stable accuracy and CGM metrics over multiple, sequential sensor cycles with no indication of degradation of sensor performance.

## Introduction

Multiple randomized controlled trials have demonstrated that the use of continuous glucose monitoring (CGM) provides superior glycemic control over standard capillary self-measurement of blood glucose (SMBG) with regard to reductions in hemoglobin A1c (HbA1c) and hypoglycemia.^1–8^ Recent studies also suggest that increases in percent time in range (%TIR, glucose values of 70–180 mg/dL) are correlated with a reduced incidence of complications such as diabetic retinopathy.^9,10^ Percent TIR has also been shown to be strongly correlated with HbA1c values across a broad range of patients, ages, and technologies.^11^

The Food and Drug Administration (FDA) approved the Eversense^®^ CGM System (Senseonics, Inc., Germantown, MD), with the first long-term, 90-day duration implantable sensor, in June 2018 for use in patients with diabetes, 18 years of age and older, based on the performance and safety results from multiple clinical trials.^12–14^ These trials demonstrated promising accuracy overall (mean absolute relative difference [MARD] against Yellow Springs Instrument [YSI] reference glucose analyzer values) of 8.5%.^12–14^ The system showed a favorable safety profile with relatively few device- or procedure-related adverse events.

The safety of the CGM system in clinical trials was corroborated in a real-world, long-term registry study of multiple sensor insertion and removal cycles.^15^ This registry demonstrated that infections and secondary procedures to remove the sensor occurred at a low rate of 2.46 and 1.90 events per 100 patient-years, respectively, and did not increase from cycle to cycle. Real-world data from first U.S. commercial users were also recently reported and demonstrated promising %TIR of 62.3% per day and mean ARD of 11.2% against fingerstick tests over a 90-day sensor cycle.^16^

The 90-day Eversense CGM System received its CE mark in May 2016 and has been available in 14 European countries and South Africa since June 2016, and subsequently, the 180-day Eversense XL CGM System received its CE mark in November 2017.

There have been no reports on the performance of the Eversense CGM System over multiple, sequential sensor insertion cycles from either real-world experience or clinical studies. This report describes the accuracy and glycemic control outcomes over multiple sensor cycles from a commercial database of patients outside of the United States.

## Methods

### Device

The Eversense CGM System and the office-based insertion procedure have been described in other reports.^12–14^ The system consists of the following components: (1) an implantable, fluorescence-based glucose sensor placed into the subcutaneous tissue of the upper arm; (2) a transmitter, which powers the sensor, held on top of the skin with a silicone-based adhesive and can be removed as desired; and (3) a mobile application for monitoring of current (real-time) and historical glucose values on a Bluetooth Low Energy-enabled device such as a smartphone. Two calibrations are required per day.

### Patient population and device use

All patients assessed in this report were real-world adult patients with diabetes for whom the Eversense CGM System was prescribed and inserted by their health care provider (HCP) across ∼1000 centers in Europe and South Africa from June 2016 to August 2019. Consistent with the device labeling at the time, the contraindications for a patient receiving a sensor were the following: planned magnetic resonance imaging during the period of sensor wear, critical illness or hospitalization, known contradiction to dexamethasone, or requirement of intravenous mannitol or mannitol irrigation solutions. Demographic information on patients was not available from the Eversense Data Management System (DMS) to ensure compliance with General Data Protection Regulation (GDPR) laws.

Sensors were to be replaced every 90 or 180 days, depending on the Eversense product used, with removal of the expired sensor and insertion of a new sensor in the opposite arm according to the device labeling. This would mean that the same area could be used for sensor placement every other cycle. However, there was no available information regarding compliance of the patient and HCP with this device labeling. The transmitter is replaced once a year.

### Data collection

The Eversense DMS is a web-based application that enables patients to upload sensor glucose (SG) readings and calibration points from the Eversense Mobile Application. As part of the Eversense CGM System design, SG data, including alerts and any events entered on the application such as glucose values other than calibration points, insulin boluses, meals, illness, and exercise, are automatically uploaded to the DMS when the transmitter is worn and the smartphone is connected to Wi-Fi or cellular networks. These data are displayed using numerous report formats, including but not limited to various %TIR outputs, and the ambulatory glucose profile to inform HCPs and users.^17^

The DMS database was used to evaluate GDPR-compliant, deidentified SG data and accuracy against SMBG from June 22, 2016, through August 3, 2019. All SG data from all users with at least four sensor cycles were included in the evaluation. As of August 3, 2019, 945 patients had at least 4 sensor cycles of data and were included in this report.

### Data analysis

Sensor accuracy was determined using paired SG and calibration SMBG values obtained using the patient's personal blood glucose meter. Calibration SMBG values were entered into the mobile application twice daily, 10–14 h apart. SG readings are not displayed if no calibration was performed in a 24-h period per system design. Mean and median ARD values were calculated using all SMBG/SG matched pairs obtained throughout each sensor cycle. Each SMBG value was paired with the corresponding CGM measurement obtained within 5 min of the entered SMBG.

The percentage of SG values either (1) within 20 mg/dL of the matched SMBG values for SMBG values <80 mg/dL or (2) within 20% of the SMBG values for SMBG values >80 mg/dL, referred to as 20/20% agreement rate, was also calculated.

Over each sequential sensor period, descriptive statistics for CGM metrics were calculated by the patient using all available SG values, including mean, standard deviation (SD), coefficient of variation (CV), and glucose management indicator (GMI; a mathematical estimate of HbA1c^18^). Finally, the percent of SG values and time (in minutes) the patient had readings in each of the following glucose ranges over the 24-h period were calculated: <54, 54 to <70, <70, 70–180, >180, >180–250, and >250 mg/dL.

The median percent transmitter wear time was calculated across each wear cycle for all 945 users included in this analysis.

## Results

### Accuracy, CGM metrics, and transmitter wear time

Table 1 summarizes the accuracy and CGM metrics from 945 users during each of the first four sensor cycles. The percentage of 180-day sensors used during each cycle increased over time as the longer-duration device was made available. The percentage of 180-day sensors from cycle 1 to cycle 4 was 9%, 39%, 68%, and 88%, respectively. These data include a total of 1269 patient-years of real-world follow-up. The average values across patients for the MARD ranged from 11.5% to 11.9% during each sensor cycle (comprising 152,206, 174,645, 206,024, and 172,587 paired points for the four respective sensor cycles). The average values for the SD ranged from 3.6% to 4.7%; the median ARD across sensor cycles ranged from 10.9% to 11.3%.

**Table 1.** Eversense Continuous Glucose Monitoring Metrics and Accuracy over Four Sequential Sensor Cycles in 945 Patients

Cycle	Sensor cycle 1	Sensor cycle 2	Sensor cycle 3	Sensor cycle 4
CGM metric	Mean (SD) [median, IQR]	Mean (SD) [median, IQR]	Mean (SD) [median, IQR]	Mean (SD) [median, IQR]
SG mean, mg/dL	156.5 (24.6) [154.3, 140.0–170.8]	158.2 (25.9) [155.9, 141.0–172.9]	157.4 (26.1) [153.9, 140.1–171.5]	156.5 (26.5) [153.8, 138.8–171.3]
SG SD, mg/dL	54.7 (12.4) [54.7, 46.8–62.9]	55.8 (12.8) [55.6, 47.4–64.7]	55.5 (12.8) [55.1, 46.6–64.3]	55.5 (13.0) [55.3, 46.3–64.7]
SG CV	0.35 (0.06) [0.35, 0.31–0.39]	0.35 (0.06) [0.36, 0.32–0.39]	0.35 (0.06) [0.36, 0.32–0.39]	0.36 (0.06) [0.36, 0.32–0.40]
GMI, %	7.04 (0.59) [6.99, 6.63–7.38]	7.08 (0.62) [7.02, 6.66–7.44]	7.06 (0.62) [6.97, 6.66–7.40]	7.04 (0.63) [6.97, 6.61–7.40]

Time in glucose range, %	Mean (SD) [median, IQR]	Time (min)	Mean (SD) [median, IQR]	Time (min)	Mean (SD) [median, IQR]	Time (min)	Mean (SD) [median, IQR]	Time (min)
<54 mg/dL	1.1 (1.2) [0.7, 0.3–1.6]	16	1.2 (1.3) [0.8, 0.3–1.7]	17	1.2 (1.3) [0.9, 0.3–1.8]	17	1.3 (1.6) [0.8, 0.3–1.7]	19
54 to <70 mg/dL	3.5 (2.7) [3.0, 1.5–4.9]	50	3.5 (2.8) [2.9, 1.6–5.0]	50	3.6 (2.6) [3.2, 1.6–4.9]	52	3.7 (3.0) [3.1, 1.6–5.1]	53
<70 mg/dL	4.6 (3.8) [3.7, 1.8–6.3]	66	4.7 (4.0) [3.7, 1.9–6.6]	68	4.8 (3.8) [4.0, 2.0–6.7]	69	5.0 (4.4) [4.1, 2.0–6.9]	72
70–180 mg/dL	64.5 (15.1) [65.3, 55.0–75.2]	929	63.2 (15.7) [64.2, 53.6–74.4]	910	63.7 (15.7) [65.1, 53.6–74.4]	917	64.0 (15.6) [65.0, 53.9–75.1]	922
>180 mg/dL	30.9 (16.1) [29.3, 19.4–41.1]	445	32.0 (16.7) [30.4, 19.8–42.2]	461	31.5 (16.8) [29.3, 19.6–41.9]	455	31.0 (16.8) [29.0, 19.0–41.6]	446
>180–250 mg/dL	22.8 (9.5) [23.4, 16.6–29.4]	328	23.2 (9.4) [23.7, 16.9–29.5]	334	22.9 (9.5) [23.3, 17.0–29.6]	330	22.4 (9.5) [22.8, 15.9–28.9]	323
>250 mg/dL	8.1 (8.4) [5.5, 2.1–11.3]	117	8.8 (9.1) [6.1, 2.2–12.6]	127	8.6 (9.2) [5.8, 2.2–12.0]	124	8.6 (9.2) [5.5, 2.2–11.8]	124

Sensor performance	Mean (SD) [median, IQR]	Mean (SD) [median, IQR]	Mean (SD) [median, IQR]	Mean (SD) [median, IQR]
Mean ARD	11.9 (3.6) [11.3, 9.8–13.2]	11.5 (4.0) [10.9, 9.4–12.9]	11.8 (4.7) [11.1, 9.2–13.2]	11.5 (4.1) [10.9, 9.3–12.8]
20/20%	83.0 (8.9) [84.5, 78.5–89.1]	83.9 (9.3) [85.7, 79.6–90.4]	82.9 (10.4) [85.2, 78.6–89.8]	83.8 (9.7) [85.2, 80.0–90.2]
Median wear time per day (%)	83.2	83.6	84.8	85.8

20/20%, The percentage of system readings within 20 mg/dL or 20% of self-monitoring of blood glucose values; ARD, absolute relative difference; CGM, continuous glucose monitoring; CV, coefficient of variation; GMI, glucose management indicator; IQR, interquartile range; SD, standard deviation; SG, sensor glucose.

The average of each individual patient's mean SG value during each sensor cycle ranged from 156.5 to 158.2 mg/dL across the four sensor cycles with a median that ranged from 153.8 to 155.9 mg/dL. The average SD values ranged from 54.7 to 55.8 mg/dL during each cycle with average CV values that ranged from 0.35 to 0.36. The average estimated HbA1c (GMI) ranged from 7.04% to 7.08%.

The mean percent of SG readings in significant hypoglycemia (<54 mg/dL) over each cycle ranged from 1.1% to 1.3% (16–19 min) per day and the average percent of SG readings <70 mg/dL ranged from 4.6% to 5.0% (66–72 min) per day. The average percent TIR ranged from 63.2% to 64.5% (910–929 min) per day. The average percent of SG readings in the hyperglycemic range (>180–250 mg/dL) ranged from 22.8% to 23.2% (328–334 min) per day. Finally, at the significant hyperglycemic range of >250 mg/dL, the average percent of readings ranged from 8.1% to 8.8% (117–127 min) per day.

The median transmitter wear time over each cycle ranged from 83.2% to 85.8% per day with an overall median of 84.1% per day (Table 1).

## Discussion

Evaluation of glycemic values from the Eversense CGM System from 945 patients with diabetes in a real-world setting showed stability of both accuracy against SMBG and CGM metrics over four consecutive sensor cycles. Across 1269 patient-years of follow-up, the device showed stable performance over multiple cycles with average MARD values in a narrow range of 11.5%–11.9% and the 20/20% metric ranging from 82.9% to 83.9%. CGM metrics were also consistent over multiple cycles with estimated HbA1c (GMI) ranging from 7.04% to 7.08%; additionally, the average CV values of 0.35–0.36 at each cycle were consistent with the results observed in the analysis of real-world data from the first 205 U.S. commercial patients.^16^ The average %TIR was constant at ∼15 h per day during each cycle, which corroborated the findings in the real-world U.S. user analysis.^16^ Time spent in the severe hypoglycemic range was uniform, ranging from 1.1% to 1.3% (16–19 min) per day. Last, the overall median transmitter wear time across the four consecutive cycles was 84.1%, consistent with the 83.6% observed in the U.S. real-world analysis.^16^

The mean TIR of 64% observed in this analysis was similar to the 63% value reported in the real-world evaluation of the first 205 U.S. users. The %TIR with the Eversense CGM System in this analysis was similar to^19^ or greater than the values reported for other commercially available CGM systems (51% TIR from the Diamond study of CGM in study participants on multiple daily insulin injections).^4,11^

The MARD values of 11.5%–11.9% across sensor cycles were higher than the 8.5% value reported in the PRECISE II pivotal trial^13^; however, this finding is not surprising due to the fact that reference glucose values in PRECISE II were in a highly controlled clinical setting against YSI rather than patients' personal blood glucose meter. Unlike in pivotal clinical trials, a mix of commercially available blood glucose meters and a variety of fingerstick practices are used from patient to patient with some using ideal fingerstick methods of washing and drying their hands before fingerstick tests, while others may use suboptimal methods (e.g., fingersticks with dirty fingers). Heinemann et al. have demonstrated that the MARD values against the reference of SMGB are higher than that for YSI across multiple CGM systems.^20^ Notably, a head-to-head evaluation of the performance of the Eversense System against two other CGM systems using the same blood glucose meter showed that there is a possibility that the Eversense System is better than the other CGM systems with similar observed MARD values to those reported in this evaluation.^21^

MARD has been shown to be influenced by the foreign body response to transcutaneous sensors.^22^ Specifically, these researchers demonstrated that the greater the number of macrophages near the sensor–tissue interface, the higher the MARD and the resultant lower accuracy. However, these data show no evidence of degradation of performance from the repeated insertion and removal procedures occurring in approximately the same subcutaneous tissue of the body.

While there was no degradation of performance, there was also no improvement in glucometrics in this patient population across multiple sensor cycles. The reasons why there were no significant differences among the multiple sensor cycles in this study are unclear. One of the potential reasons is that the benefit of CGM appeared to be early, during the first sensor wear, when glucose control over the 90- or 180-day time period already approached most of the glucometric targets previously described.^23^ This is similar to what has been seen previously by Bergenstal et al.,^24^ where the benefit of CGM was seen by the first observation at 3 months and sustained, not improved, over the 12-month study period. Another potential reason for no difference in metrics across cycles is that many of the patients had used CGM before, already availing themselves of this tool for diabetes management.

One of the primary benefits of the implantable Eversense CGM System compared with other CGM systems is its long-term duration of 90 or 180 days. Other commercially available CGM systems, which are inserted transcutaneously, last no longer than 14 days. In a 6-month time period, the 180-day Eversense System is inserted and removed once, whereas a short-term 14-day device would need to be inserted and removed 12 times. The office-based insertion and removal procedure on average takes <5 to 10 min and only requires local injection of lidocaine and a small 5-mm incision that is closed without sutures.

Patients have shown their preference for Eversense in two prior clinical studies, where surveys showed that over 80% of patients, 45% of whom had used other CGM systems, wanted to be reinserted with another sensor, demonstrating the acceptance and usability of the Eversense System.^14,25^ In free-form responses using the CGM satisfaction scale, many patients cited freedom from having to replace the CGM weekly as a positive attribute.^14^ In addition, in a large registry of the long-term safety of Eversense over multiple sensor insertion and removal cycles, only 11% of patients had discontinued the Eversense System by the last follow-up reported, with the predominant reason for discontinuation cited as lack of medical reimbursement or temporary discontinuation of prescription order.^15^

While all CGM systems carry risks, those associated with the Eversense System were not serious, relatively infrequent, and manageable. Infection related to the procedure was reported as the most frequently occurring risk, which occurred at a rate of <1%, in a large registry study of multiple insertion and removal cycles in over 3000 patients.^15^ For a 180-day sensor, a 1% rate of infection would equate to an infection occurring once in 50 years of use. Importantly, the infection rate with Eversense was shown to be three to five times lower than that reported with insulin infusion pumps.^15^ Therefore, given the stable performance across cycles reported here, low rate of risks reported previously, and preference for Eversense shown in the majority of patients in previous studies, the benefits of Eversense outweigh the risks and provide patients with a long-term implantable CGM system that many prefer over short-term transcutaneous systems.

Limitations of this report are that only four sensor cycles were evaluated in a CGM system that is intended to be used over many years, and characteristics of this patient population are unknown other than that they have diabetes. However, the relatively large number of patients included in the analysis with ∼1270 patient-years of sensor use and an average follow-up exceeding 1 year without any signal of degradation in performance are encouraging. Another limitation is that the SMBG values that were used to determine accuracy are entered manually by patients, which may lead to inadvertent or intentional entry errors.

In summary, this report demonstrates that the accuracy and CGM metrics with the Eversense CGM System are stable over multiple sensor insertion and removal cycles in real-world use. No degradation of performance from the insertion and removal procedures and resultant tissue response to the sensor were observed. The stable long-term performance in the present evaluation, considered in context of the favorable long-term safety profile described in previous reports, provides substantial real-world evidence that the Eversense CGM System is a helpful tool to support patients in managing their diabetes in the long term.
